# Research progress on active ingredients of traditional Chinese medicine in the treatment of asthenozoospermia

**DOI:** 10.3389/frph.2026.1837460

**Published:** 2026-04-23

**Authors:** Hongyu Wang, Haichao Ge, Chunyan Ge, Lianwen Zheng, Lianhai Jin, Donghai Zhao

**Affiliations:** 1 Clinical Skills Experiment Center, Jilin Medical University, Jilin, China; 2 Department of Gastroenterology, the First Affiliated Hospital of Ningbo University, Ningbo, Zhejiang, China; 3 Department of Pediatric Surgery, Taizhou Hospital, Taizhou, Zhejiang, China; 4 Department of Obstetrics and Gynecology, the Second Hospital of Jilin University, Changchun, Jilin, China; 5 College of Basic Medicine, Jilin Medical University, Jilin, China

**Keywords:** antioxidant stress, asthenozoospermia, multi-target therapy, reproductive pharmacology, traditional Chinese medicine extracts

## Abstract

Asthenozoospermia, a main cause of male infertility, is characterized by diminished sperm motility and increased morphological abnormalities, presenting a major barrier to natural conception. While current treatments provide limited benefit, their effectiveness is often variable, invasive, and costly. In contrast, Traditional Chinese Medicine (TCM), guided by holistic principles and syndrome differentiation, offers a distinct therapeutic approach. Through multi-component, multi-target, and multi-pathway actions, TCM herbal compounds demonstrate considerable potential to improve sperm quality and address core pathological mechanisms of asthenozoospermia. This review systematically summarizes the therapeutic potential of four major classes of TCM active ingredients—flavonoids, polysaccharides, saponins, and phenols—in the management of asthenozoospermia. We elucidate their underlying molecular mechanisms, particularly focusing on the modulation of oxidative stress and inflammatory pathways. Furthermore, this review discusses current limitations of TCM in this area, addresses a knowledge gap regarding TCM active components for asthenozoospermia, and offers a reference for future research.

## Graphical Abstract

**Figure:**
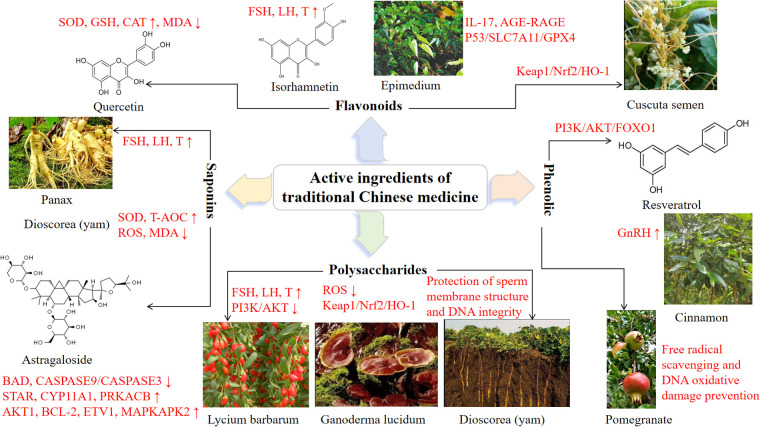

## 1 Introduction

Male infertility poses substantial medical and socioeconomic challenges, affecting an estimated 15% of couples worldwide (1–3). Asthenozoospermia, characterized by diminished sperm motility, represents a predominant factor, accounting for approximately 82% of male infertility cases. This condition is frequently associated with concomitant reductions in sperm count and/or abnormalities in morphology—termed oligozoospermia and teratozoospermia, respectively (2). Asthenozoospermia is clinically defined by a decrease or absence of motile spermatozoa in semen. Based on World Health Organization criteria, the diagnosis is established when the total sperm motility falls below 40% and the proportion of progressive motile sperm is less than 32% (2–4). Impaired sperm motility represents the core pathological defect in this condition, which significantly compromises the ability of sperm to reach and fertilize the oocyte, ultimately leading to fertilization failure (5). Furthermore, spermatozoa from asthenozoospermic patients typically exhibit a markedly elevated apoptosis rate, alongside significantly reduced proliferative capacity and motility compared to normal controls (6). The etiology of asthenozoospermia is multifactorial and remains incompletely elucidated in a substantial proportion of cases. Commonly associated factors encompass cryptorchidism, varicocele, reproductive tract infections, endocrine disturbances, exposure to environmental toxicants, iatrogenic causes (e.g., chemotherapy), suboptimal lifestyle habits, and genetic predispositions (2–4). Alongside genetic and pathological causes, environmental and behavioral factors constitute key contributors to the development and progression of asthenozoospermia. Detrimental dietary patterns, particularly the consumption of ultra-processed foods (UPFs)—high in added sugars, total and saturated fats but low in protein, fiber, and micronutrients—are positively associated with the risk of asthenozoospermia (7). Furthermore, the intake of specific animal-derived monounsaturated fatty acids (MUFAs), such as stearic, palmitoleic, and arachidonic acids, is also linked to impaired sperm quality and an increased incidence of this condition (8). Beyond dietary factors, sleep quality represents another significant modifiable risk factor for asthenozoospermia. Studies indicate that men experiencing sleep disturbances face a markedly higher risk of developing this condition compared to those with healthy sleep patterns (9). Iatrogenic interventions, particularly certain chemotherapeutic agents, constitute another important etiology. Drugs such as cyclophosphamide can induce testicular tissue damage and disrupt endocrine homeostasis, notably reducing serum testosterone levels. These perturbations often lead to spermatogenic dysfunction, manifesting as oligozoospermia or even azoospermia (10).

**Table 1:** The mechanism of traditional Chinese medicine active ingredients in treating asthenozoospermia.

Active ingredients of TCM	TCM	Mechanism	References
Flavonoids	Quercetin	SOD, GSH, CAT ↑, MDA ↓	(17, 18)
	Isorhamnetin	FSH, LH, T ↑	(17, 19)
	Epimedium	Regulation of the IL-17, AGE-RAGE, P53/SLC7A11/GPX4 signaling pathways	(24, 27)
	Cuscuta semen	Regulation of the Keap1/Nrf2/HO-1 signaling pathway	(29)
Polysaccharides	Lycium barbarum	FSH, LH, T ↑, PI3K/AKT ↓	(35, 36)
	Ganoderma lucidum	ROS ↓, Regulation of the Keap1/Nrf2/HO-1 signaling pathway	(39)
	Dioscorea (yam)	Protection of sperm membrane structure and DNA integrity	(41)
Saponins	Panax	FSH, LH, T ↑	(35)
	Dioscorea (yam)	SOD, T-AOC ↑, ROS, MDA ↓	(46)
	Astragaloside	AKT1, BCL-2, ETV1, MAPKAPK2 ↑ STAR, CYP11A1, PRKACB ↑ BAD, CASPASE9/CASPASE3 ↓	(48)
Phenolic	Resveratrol	Regulation of the PI3K/AKT/FOXO1 signaling pathway	(52)
	Cinnamon	GnRH ↑	(53, 54)
	Pomegranate	Free radical scavenging and DNA oxidative damage prevention	(53, 55)
Combination	Lycium barbarum and Panax	ROS, MDA ↓, GSH-Px ↑ Regulation of the ZNT3 signaling pathway	(35)

TCM, traditional Chinese medicine; SOD, superoxide dismutase; GSH, glutathione; CAT, catalase; MDA, malondialdehyde; FSH, follicle-stimulating hormone; LH, luteinizing hormone; T, testosterone; ROS, reactive oxygen species; GSH-Px, glutathione peroxidase; T-AOC, total antioxidant capacity; FOXO1, forkhead box protein O1; GnRH, gonadotropin-releasing hormone.

Currently, the clinical management of asthenozoospermia primarily relies on antioxidant therapy and lifestyle modification. While these approaches can partially improve certain semen parameters, their efficacy in addressing oligozoospermia remains limited (11). Research over recent decades has predominantly explored hormone regulation, antioxidant supplementation, enhancement of energy metabolism, trace element replenishment, and assisted reproductive technologies (ART). Although these strategies have enabled successful pregnancies for many couples and demonstrated measurable benefits, they are often associated with significant drawbacks, including high cost, procedural invasiveness, potential adverse effects, and other clinical risks (12). In contrast to conventional approaches, traditional Chinese medicine has demonstrated unique therapeutic advantages in the long-term clinical management of male infertility (13). This review therefore systematically synthesizes recent evidence on traditional Chinese medicine and its active ingredients for asthenozoospermia, identifies current limitations, and proposes future research avenues, aiming to inform and advance traditional Chinese medicine-based interventions in this field.

## 2 The role of traditional Chinese medicine based on flavonoids in anti-asthenozoospermia

Flavonoids are widely distributed plant secondary metabolites. Their basic scaffold is a C6-C3-C6 biphenyl-pyran structure, in which two benzene rings (A and B) are linked via an oxygen-containing heterocycle (ring C). Depending on the oxidation state, substituents, and degree of unsaturation of ring C, flavonoids are further divided into subclasses such as flavanols and flavonols. Their physicochemical properties vary with pH (14). Traditional extraction methods—like leaching and Soxhlet extraction—suffer from long processing times, high energy use, and excessive organic solvent consumption. In contrast, ultrasonic-assisted extraction has emerged as a promising green alternative (15). Flavonoids display a broad range of pharmacological effects, including antioxidant, anti-inflammatory, antitumor, and cardiovascular protective activities (14–16). These properties provide a strong rationale for their use in treating asthenozoospermia, a condition closely tied to oxidative stress and inflammation. Among flavonoids, quercetin and isorhamnetin are two extensively studied representative compounds (17). Quercetin, abundantly found in traditional Chinese medicine herbs such as Plantago asiatica and raspberries, has demonstrated well-established anti-inflammatory and antioxidant properties across various experimental models. Research indicates that quercetin can thicken the seminiferous epithelium, increase the population of spermatogenic cells, lower the sperm DNA fragmentation index, and significantly enhance testicular sperm density and motility. Regarding its antioxidant mechanism, quercetin elevates the levels of key antioxidant enzymes and molecules—including superoxide dismutase (SOD), glutathione (GSH), and catalase (CAT)—in spermatozoa, while concurrently reducing the content of the lipid peroxidation marker malondialdehyde (MDA). This action collectively strengthens the sperm's endogenous antioxidant defense capacity (17, 18). Isorhamnetin also exhibits potent free radical scavenging capacity, effectively mitigating spermatogenic dysfunction induced by oxidative stress. Furthermore, this compound stimulates the secretion of key reproductive hormones—follicle-stimulating hormone (FSH), luteinizing hormone (LH), and testosterone (T)—thereby improving the spermatogenic microenvironment through modulation of the hypothalamic-pituitary-gonadal axis (17, 19). Mechanistically, the study by Ye QN et al. demonstrated that the core targets of Wuzi Yanzong Pill in the context of oligoasthenospermia are primarily enriched in biological processes related to hormone response, steroid hormone response, and oxidative stress. Although this formula is a multi-component preparation, network pharmacology analysis revealed that its top five key active constituents (ranked by degree) are quercetin, kaempferol, *β*-sitosterol, isorhamnetin, and stigmasterol, indicating that flavonoids likely play a major role in its overall therapeutic effect. Nevertheless, the specific contribution and comparative potency of individual bioactive components in improving asthenozoospermia remain to be fully elucidated. Furthermore, the study suggests that Wuzi Yanzong Pill may exert its pharmacological actions by modulating several signaling pathways, including IL-17, HIF-1, PI3K/AKT, and AGE/RAGE (17). Similarly, Zhang ZH et al. confirmed these findings in a study exploring the mechanism of Wuzi Yanzong Formula in a mouse model of oligoasthenospermia (20).

Epimedium is a commonly used tonic herb in traditional Chinese medicine, known for its diverse pharmacological activities, including anti-inflammatory, antioxidant, and sexual function-enhancing effects (21). These benefits are largely attributed to its flavonoid components (FEE), which are considered the primary bioactive constituents of the herb (22, 23). Research indicates that Epimedium and its bioactive components can promote spermatogenesis, improve sperm motility, protect testicular structure, and regulate the serum levels of FSH, LH, and T (24, 25). Mechanistically, the study by Li YR et al. demonstrated that the therapeutic effect of Epimedium on asthenozoospermia involves the modulation of signaling pathways such as IL-17 and AGE/RAGE (24). Furthermore, emerging evidence highlights the significant role of ferroptosis—a form of regulated cell death driven by iron overload, antioxidant system failure, and lipid peroxidation—in the pathogenesis of asthenozoospermia and its associated impairment of testicular spermatogenesis (26). In this context, Jiang XC et al. demonstrated that Epimedium can alleviate testicular ferroptosis by regulating the P53/SLC7A11/GPX4 axis, thereby improving spermatogenic function and sperm quality (27).

Cuscuta semen, a traditional tonic herb in Chinese medicine, is historically prescribed for its renal-tonifying and essence-replenishing properties, as well as for reducing spermatorrhea, polyuria, preventing miscarriage, and improving vision. It is commonly used in clinical practice to manage various reproductive disorders in both men and women. Modern studies indicate that flavonoids represent a key group of bioactive constituents responsible for its reproductive protective effects (28). Feng JL et al. demonstrated that flavonoids from Cuscuta semen regulate the synthesis and release of sex hormones, promote spermatogenesis, and alleviate pathological damage to testicular tissue in rat models, leading to significantly increased sperm concentration and total motility. Mechanistically, the study revealed that Cuscuta semen enhances antioxidant capacity and protects testicular spermatogenic function by activating the Keap1/Nrf2/HO-1 signaling pathway (29).

The aforementioned studies confirm that flavonoid-rich active components from traditional Chinese medicine exert their therapeutic effects through a multi-target approach encompassing antioxidative stress, hormonal regulation, and modulation of key signaling pathways. This integrated mechanism systematically elucidates the modern pharmacological basis of herbal formulations such as Wuzi Yanzong Pills and herbs including Epimedium and Cuscuta in improving asthenozoospermia. Such convergent pharmacological actions not only provide a scientific foundation for the documented efficacy of these traditional Chinese medicine interventions but also establish a solid basis for developing innovative, evidence-based traditional Chinese medicine therapies for asthenozoospermia through further active ingredient discovery and mechanistic research. However, the studies discussed above have several limitations. For example, when examining the anti-asthenozoospermia effects of flavonoids such as quercetin and isorhamnetin, most reports only measured oxidative markers like SOD and MDA, without probing deeper into relevant signaling pathways or specific molecular targets,such as Keap1 and AMPK. Future work should therefore clarify the precise molecular mechanisms involved. Moreover, the current conclusions rest entirely on animal and cell experiments; the potential toxicity of these TCM components has not been clearly characterized. Whether these findings translate to humans remains unknown and will require extensive clinical testing to establish safety.

## 3 The role of traditional Chinese medicine based on polysaccharides in the treatment of asthenozoospermia

Polysaccharides are macromolecular polymers formed by more than ten monosaccharide units linked via glycosidic bonds, and are widely distributed in animals, plants, and microorganisms. Based on monosaccharide composition, they can be classified into homopolysaccharides (e.g., starch) and heteropolysaccharides (e.g., gum arabic). These compounds generally exhibit poor water solubility, lack sweetness, and are non-reducing. Common extraction techniques include hot-water extraction, acid- or alkali-assisted extraction, and enzymatic extraction. Bioactive polysaccharides possess diverse physiological activities such as immunomodulatory, anti-inflammatory, antioxidant, hypoglycemic, and lipid-lowering effects, demonstrating significant potential in both food and pharmaceutical applications (30–32). In recent years, polysaccharides have gained increasing attention for their protective role in the reproductive system, offering novel therapeutic candidates and research perspectives for the treatment of asthenozoospermia.

Lycium barbarum contains multiple bioactive compounds, with Lycium barbarum polysaccharides (LBP) being the main active constituents. LBP exhibit antioxidant, anti-inflammatory, neuroprotective, and immunomodulatory activities (33, 34). In a mouse model of asthenozoospermia induced by Tripterygium wilfordii polyglycoside (GTW), Li XR et al. found that LBP treatment increased testicular and epididymal indices, reduced tissue damage, improved sperm quality, and raised serum FSH, LH, and T levels. These results suggest LBP has therapeutic potential against asthenozoospermia (35). Separately, in a streptozotocin-induced rat model of diabetic testicular dysfunction, Shi GJ et al. showed that LBP mitigated abnormal autophagy via the PI3K/AKT pathway, enhanced antioxidant capacity, and improved testicular function and sperm quality (36). Although that study did not use an asthenozoospermia model, the observed protection against testicular dysfunction further supports the reproductive benefits of LBP.

Ganoderma lucidum has long been used as both a functional food and a medicinal fungus in traditional practice. Its bioactivities-anti-inflammatory, antioxidant, antitumor, and immunomodulatory-derive from a range of active compounds (37, 38). Among them, Ganoderma lucidum polysaccharide (GLP), a major water-soluble fraction, is known for hepatoprotective, immunoregulatory, antioxidant, and antitumor effects (39). In an oligospermia mouse model induced by adenine, Hao Y. et al. found that GLP treatment significantly lowered testicular ROS levels. This was accompanied by increased sperm count, density, and motility, along with higher epididymal and testicular indices. Histopathology showed less testicular damage. Mechanistically, these benefits were linked to modulation of the Keap1/Nrf2/HO-1 signaling pathway (39).

As a medicinal and culinary plant, Dioscorea (commonly called yam) offers considerable therapeutic promise. Its diverse repertoire of bioactive compounds-including polysaccharides, steroidal saponins, flavonoids, and amino acids-has underpinned traditional uses against diabetes, diarrhea, and asthma. Among these constituents, yam polysaccharide (YP) stands out. Composed of mannose, xylose, arabinose, glucose, and galactose, YP exhibits a range of biological effects, such as antioxidant, anti-inflammatory, and antitumor activities (40, 41). In an *in vitro* study by Zhang MH et al., YP treatment safeguarded sperm membrane structure and DNA integrity, leading to enhanced sperm motility and prolonged survival time. Notably, these protective effects on sperm function followed a clear dose-dependent pattern (41).

In summary, polysaccharides exhibit considerable therapeutic potential against asthenozoospermia through multifaceted mechanisms. These include the regulation of reproductive endocrinology, activation of endogenous antioxidant pathways (e.g., Keap1/Nrf2/HO-1), maintenance of autophagy homeostasis, and direct protection of sperm structure and function. Future research should focus on elucidating the structure-activity relationships, precise cellular targets, and synergistic mechanisms of polysaccharides within complex formulations to facilitate their translation into clinical therapies. Similarly, polysaccharide compounds suffer from the same shortcoming—only animal and cell data are available, with no toxicity assessment—so whether they can be used directly in humans remains unclear.

## 4 The role of traditional Chinese medicine based on saponins in the treatment of asthenozoospermia

Saponins represent a structurally diverse class of glycosides widely distributed in medicinal plants, including soybean, Astragalus, ginseng, and Bupleurum. Based on the structure of the aglycone moiety, saponins are broadly classified into two major groups: steroidal saponins, predominantly found in monocotyledons, and triterpenoid saponins, mainly derived from dicotyledons. Their molecules consist of a hydrophilic sugar chain linked via glycosidic bonds to a hydrophobic aglycone, conferring distinct amphiphilic properties. Commonly employed extraction methods include maceration and Soxhlet extraction (42, 43). Studies have shown that saponins exhibit a wide spectrum of pharmacological properties, including anti-inflammatory, immunomodulatory, antifibrotic, and antitumor activities, and demonstrate promising potential in the protection of the reproductive system (42, 43).

Panax, a widely used traditional Chinese medicine, exhibits broad pharmacological activities. Its primary bioactive constituents, ginsenosides, are classified into two major types based on their aglycone structure: protopanaxadiol (PPD) and protopanaxatriol (PPT). Research has confirmed that these compounds possess anti-inflammatory, antioxidant, and neuroprotective properties (35, 44). Li XR et al. utilized both a GTW-induced mouse model of asthenozoospermia and a hydrogen peroxide-exposed GC-2 cell model in their study. Their findings indicated that ginsenoside Rb1 (GRb1) significantly increased testicular and epididymal indices, improved sperm quality, and elevated serum levels of FSH, LH, and T (35). Further mechanistic investigation revealed that the combined application of GRb1 and LBP markedly reduced the levels of ROS and MDA in testicular tissues and GC-2 cells, while enhancing the activity of glutathione peroxidase (GSH-Px), thereby strengthening the overall antioxidant defense capacity (35). Zinc, an essential trace element, plays a vital role in hormone synthesis and spermatogenesis, particularly in supporting sperm motility (45). The study further demonstrated that the combined intervention of GRb1 and LBP alleviated oxidative stress by inhibiting the zinc transporter (ZNT3) signaling pathway and increasing the total zinc content in testicular tissue, thereby counteracting asthenozoospermia (35).

In addition to its polysaccharides, Dioscorea contains diosgenin-a steroidal saponin known to modulate the endogenous synthesis of steroid hormones (e.g., sex hormones and corticosteroids). This hormonal effect underpins its beneficial role in improving sperm activity (46). Zhou X et al. employed a cyclophosphamide-induced mouse model of asthenozoospermia. Their study demonstrated that diosgenin intervention significantly increased epididymal and testicular indices, improved key semen quality parameters, enhanced the activity of SOD and total antioxidant capacity (T-AOC), and reduced the levels of MDA and ROS. These findings collectively suggest that diosgenin exerts its potential therapeutic effect against asthenozoospermia primarily by bolstering the endogenous antioxidant defense system (46).

Astragaloside (AST), a principal bioactive triterpenoid saponin derived from Astragalus, possesses a broad spectrum of pharmacological properties, including antioxidant, anti-apoptotic, anti-inflammatory, and immunomodulatory activities (47, 48). Using a cyclophosphamide-induced mouse model of asthenozoospermia, Fan QG et al. demonstrated that AST ameliorates reproductive impairment through multi-pathway mechanisms. First, it modulates cell survival and proliferation by upregulating anti-apoptotic (e.g., AKT1, BCL-2) and proliferation-related genes (e.g., ETV1, MAPKAPK2), while downregulating pro-apoptotic genes such as BAD and CASPASE9/CASPASE3. Second, AST enhances local testosterone synthesis in the testes by increasing the expression of key steroidogenic proteins (STAR, CYP11A1, PRKACB). These combined actions result in increased sperm concentration, motility, and viability, along with restoration of testicular tissue structure and function (48). These findings collectively demonstrate the multitarget and multipathway regulatory capacity of AST in spermatogenesis, providing critical experimental support for traditional Chinese medicine-derived active components as a strategic approach to treating asthenozoospermia.

Current evidence demonstrates that saponin constituents-including ginsenoside Rb1, diosgenin, and astragaloside-ameliorate asthenozoospermia via a multitarget, multilevel mechanism. This integrated action involves the regulation of hormonal balance, enhancement of antioxidant defenses, and precise modulation of testicular cell apoptosis and proliferation. These findings further suggest that combining saponins with other bioactive classes (e.g., polysaccharides) may yield synergistic therapeutic effects, a promising direction warranting systematic investigation. Research into saponin compounds against asthenozoospermia has been limited to animal and cell models, whether these compounds are biologically safe will require much more investigation.

## 5 The role of traditional Chinese medicine based on phenolic compounds in anti-asthenozoospermia

Phenolic compounds are a class of naturally occurring plant metabolites widely distributed across plant tissues. Chemically characterized by at least one aromatic ring bearing two or more hydroxyl groups, they often exhibit an amphiphilic nature. Based on structural complexity, phenolics are categorized into simple phenols and polyphenols, encompassing diverse subtypes such as phenolic acids, glycosides, terpenoids, and their derivatives (49, 50). These compounds possess a broad spectrum of pharmacological activities, including antioxidant, anti-inflammatory, antibacterial, and metabolic regulatory effects. Such multifaceted bioactivity underpins their significant potential in preventing and managing various diseases, such as cardiovascular disorders and cancer (49, 50). Recently, phenolic compounds have emerged as promising agents for enhancing male reproductive function, especially in mitigating oxidative stress-related asthenozoospermia, thereby providing valuable natural product-derived candidates for its treatment.

Resveratrol is a natural polyphenolic compound primarily sourced from plants such as raspberries and Polygonum knotweed. It exhibits a range of pharmacological properties, including antioxidant, anti-inflammatory, anti-apoptotic, and metabolic regulatory activities (51, 52). Wu CY et al. investigated resveratrol in a busulfan-induced mouse model of asthenozoospermia. Their results showed that resveratrol intervention effectively mitigated the loss of spermatogonial stem cells and promoted the recovery of spermatogenic function. Mechanistically, this protective effect is linked to the modulation of forkhead box protein O1 (FOXO1). Resveratrol likely activates the upstream PI3K/AKT signaling pathway, thereby attenuating FOXO1-mediated apoptosis and oxidative stress, which ultimately improved sperm motility and viability in the model mice (52).

Cinnamon is both a common culinary spice and a traditional Chinese medicinal material, rich in polyphenols such as catechins, proanthocyanidins, trans-cinnamic acid, and cinnamaldehyde. These constituents exhibit a broad spectrum of pharmacological activities, including antioxidant, anti-inflammatory, hypoglycemic, and neuroprotective effects. Studies have shown that cinnamon can reduce sperm abnormalities in animal models and ameliorate declines in sperm parameters induced by various toxicants (e.g., naloxone, carbon tetrachloride, lead acetate). Its main active component, cinnamaldehyde, promotes nitric oxide (NO) release, which acts on the hypothalamic-pituitary-gonadal axis to stimulate gonadotropin-releasing hormone (GnRH) secretion. This in turn enhances testosterone synthesis by testicular Leydig cells-a hormone crucial for spermatogenesis (53, 54). Although most studies report protective effects of cinnamaldehyde on human health—including blood glucose reduction (55), antitumor activity (56), and reproductive system protection (57)—this does not guarantee its absolute safety. Yun JW et al. found that high-dose intake of cinnamaldehyde extract exhibited potential nephrotoxicity and hepatotoxicity in both male and female subjects (58). This finding underscores the need to consider potential toxicity when using traditional Chinese medicine components. While the direct application of cinnamon polyphenol extract for treating asthenozoospermia has not yet been systematically investigated, its potential to support spermatogenic function is supported by preliminary evidence and warrants further in-depth exploration.

Pomegranate is rich in phenolic constituents, including tannins (e.g., ellagitannins like punicalagin), flavonoids (such as flavonols, proanthocyanidins, and anthocyanins), and phenolic acids (e.g., gallic, ellagic, and caffeic acids). The peel, in particular, is a concentrated source of tannins and a major contributor to its bioactivity. These polyphenolic compounds exert potent antioxidant and anti-inflammatory activities, enabling them to effectively target oxidative stress and chronic low-grade inflammation. Research indicates that key phenolic components in pomegranate, such as ellagic acid, can ameliorate semen quality and related biochemical parameters, exerting reproductive protective effects through free radical scavenging and prevention of DNA oxidative damage (53, 59). Although systematic clinical studies are lacking, preclinical evidence indicates that pomegranate polyphenol extract can significantly improve semen parameters, supporting its prospective utility in managing asthenozoospermia.

Current evidence indicates that phenolic compounds exert protective effects against asthenozoospermia through a dual-mode mechanism combining direct cellular protection (anti-apoptotic, antioxidant) and systemic endocrine regulation. Among them, resveratrol has been relatively well-characterized and is known to modulate apoptosis and oxidative stress via pathways such as PI3K/AKT/FOXO1. Polyphenols derived from cinnamon, pomegranate, and other botanicals represent a promising yet underexplored library of natural lead compounds. Future efforts should prioritize specific phenolic monomers—such as cinnamaldehyde and ellagic acid—for systematic pharmacodynamic evaluation and mechanistic elucidation in asthenozoospermia models, thereby accelerating their translation into clinical applications. Cinnamaldehyde's liver and kidney toxicity has been noted, but its effects on other systems, as well as the toxicities of other compounds, have yet to be studied. Clinical application of these agents thus awaits more extensive research.

## 6 The role of other potential active ingredients of traditional Chinese medicine in anti-asthenozoospermia

Previous studies have confirmed that several typical bioactive compounds from traditional Chinese medicine—flavonoids, polysaccharides, saponins, and phenols—exert a range of pharmacological effects, including anti-inflammatory and antioxidant activities. These findings have laid a solid pharmacological groundwork for exploring their potential in treating asthenozoospermia. On this basis, such active ingredients have been widely employed in animal and cell experiments to systematically evaluate their therapeutic efficacy against asthenozoospermia. Beyond these four well-recognized classes of compounds, other TCM-derived constituents—such as carotenoids, alkaloids, and coumarins—also show promise and merit further investigation.

### 6.1 The role of traditional Chinese medicine based on carotenoids in anti-asthenozoospermia

Crocin (molecular formula C44H64O24) is a naturally occurring water-soluble carotenoid derived from saffron. Its unique physicochemical properties have led to widespread use in both pharmaceutical and food industries. Modern pharmacological studies have demonstrated a range of therapeutic effects, including anti-inflammatory and antioxidant activities (60, 61). In an animal model of testicular injury induced by ionizing radiation, El-Sheikh MM et al. observed marked increases in oxidative stress markers—such as thiobarbituric acid reactive substances (TBARS), ROS, and NOx—alongside significant decreases in antioxidant indicators including GSH and catalase (CAT). Crocin administration effectively reversed these alterations. Mechanistically, these benefits were linked to modulation of the FOXO/AKT signaling pathway (61).

### 6.2 The role of traditional Chinese medicine based on alkaloids in anti-asthenozoospermia

Berberine, a bioactive alkaloid derived from plants such as Berberis, exhibits well-documented pharmacological effects including antioxidant, anti-inflammatory, lipid-lowering, and hypoglycemic activities (62, 63). In a diabetic rat model induced by streptozotocin, Song JY et al. observed elevated ROS levels in testicular tissue, which subsequently activated the JAK2/NF-*κ*B signaling pathway and increased testicular cell apoptosis and damage. Berberine treatment effectively attenuated these effects by suppressing the ROS/JAK2/NF-*κ*B pathway (63).

### 6.3 The role of traditional Chinese medicine based on coumarins in anti-asthenozoospermia

Umbelliferone (UMB) is a natural coumarin derivative found in plants of the Apiaceae family and possesses potent antioxidant, anti-inflammatory, and anti-apoptotic properties. Using a lead acetate-induced rat model of testicular damage, Hejazi M et al. found that lead exposure significantly reduced sperm quality, antioxidant enzyme levels, testosterone expression, and Bcl-2 expression, while increasing MDA, pro-inflammatory cytokines, and Bax levels. UMB treatment markedly reversed these changes. Mechanistically, UMB alleviated lead-induced testicular toxicity by reducing oxidative stress, inflammation, and apoptosis, thereby improving sperm quality and testosterone levels (64).

The studies described above indicate that non-classical bioactive compounds from traditional Chinese medicine—such as crocin, berberine, and umbelliferone—can effectively protect testicular tissue and improve sperm quality through antioxidant, anti-inflammatory, and anti-apoptotic mechanisms, acting via specific signaling pathways (e.g., FOXO/AKT, JAK2/NF-*κ*B). Although none of these agents have yet been tested in asthenozoospermia models, nor has their biosafety been fully evaluated, these findings nonetheless expand the material basis of traditional Chinese medicine for treating asthenozoospermia and offer lead compounds for developing new therapies.

## 7 Prospect

Asthenozoospermia is a major pathological cause of male infertility. Its pathogenesis not only directly impairs sperm motility and fertilization potential but also profoundly affects patients’ psychological well-being, interpersonal relationships, and overall quality of life. This review synthesizes recent advances in the therapeutic roles and molecular mechanisms of key traditional Chinese medicine active ingredients—including flavonoids, polysaccharides, saponins, and phenolic compounds—in the prevention and treatment of asthenozoospermia. These active constituents improve sperm quality and spermatogenic function by mitigating oxidative stress, modulating reproductive hormones, suppressing abnormal apoptosis, and regulating critical pathways (e.g., Nrf2/HO-1, PI3K/AKT). This multi-faceted action confirms the characteristic traditional Chinese medicine paradigm of “multiple components, multiple targets, and multiple pathways,” highlighting its integrated therapeutic profile.

Despite considerable progress, notable scientific and translational gaps remain in the application of traditional Chinese medicine for the prevention and treatment of asthenozoospermia. The following key issues require further investigation and resolution: 1. Limited scope of resource and active ingredient discovery: current research remains largely focused on a few classical herbs such as Epimedium, Lycium barbarum, and ginseng, leaving a vast array of potential traditional Chinese medicine resources systematically unexplored, such as Crocin, berberine and Umbelliferone. In the future, technologies such as bioinformatics should be integrated to discover novel active molecules with potential in combating asthenozoospermia, further enriching the pool of therapeutic candidate drugs. 2. Insufficient safety and toxicology assessment: While most traditional Chinese medicine ingredients originate from natural sources, their potential reproductive toxicity, bioaccumulation, and long-term effects on offspring development require systematic evaluation. The majority of studies reviewed herein focus on efficacy validation, with comparatively limited attention to safety profiles. Future research should establish a comprehensive assessment framework integrating toxicology and metabolomics, thereby providing a scientific basis for the safe clinical use of these agents. 3. Mechanistic research lacks systematic depth: Current studies predominantly focus on the efficacy of single compounds or herbs (e.g., ginsenosides, diosgenin), with mechanistic analysis often confined to isolated signaling pathways or molecular targets. Moving forward, integrative approaches combining multi-omics, gene editing, and artificial intelligence-enhanced network pharmacology should be employed. These tools can systematically map the multidimensional regulatory networks through which traditional Chinese medicine formulas or active-ingredient combinations influence the entire spermatogenic cascade—from germ cell development and maturation to sperm capacitation and motility. Such efforts will yield a more comprehensive and profound scientific foundation for deciphering the pathology of asthenozoospermia and guiding rational drug development. 4. Clinical translation and evidence-based research require acceleration: The majority of current investigations remain confined to animal and cellular models, with a notable scarcity of robust clinical evidence—particularly from large-sample, multicenter, randomized, double-blind trials. Future efforts must therefore prioritize translational studies, and establish asthenozoospermia animal models and clinical evaluation frameworks that align with traditional Chinese medicine syndrome differentiation, thereby facilitating the systematic transition of traditional Chinese medicine interventions from experimental validation to clinically effective application.

Traditional Chinese medicine and its bioactive constituents present distinctive strengths in the multi-target, holistic management of asthenozoospermia, with considerable translational promise. To address existing challenges, future work must center on clinical needs, mechanistic depth, and practical application. A concerted strategy—encompassing rigorous mechanistic research, robust clinical validation, drug delivery innovation, and synergy exploration—is needed to enable the transition from empirical use to precision therapy, thereby offering improved and personalized treatment options for global patients.
